# Integrative Functional Genomics Analysis of Kaposi Sarcoma Cohorts

**DOI:** 10.21203/rs.3.rs-6146471/v1

**Published:** 2025-03-11

**Authors:** Ezequiel Lacunza, Valeria Fink, Julian Naipauer, María E. Salas, Ana M. Gun, Mary J. Goldman, Jingchun Zhu, Sion Williams, María I. Figueroa, Pedro Cahn, Omar Coso, Ethel Cesarman, Juan C. Ramos, Martín C. Abba

**Affiliations:** CINIBA, Universidad Nacional de La Plata; Dirección de Investigaciones, Fundación Huésped, Buenos Aires, Argentina.; Instituto de Fisiología (IFIBYNE), Biología Molecular y Neurociencias (IFIBYNE), Buenos Aires, Argentina; CINIBA, Universidad Nacional de La Plata; Dirección de Investigaciones, Fundación Huésped, Buenos Aires, Argentina.; UC Santa Cruz Genomics Institute, University of California; UC Santa Cruz Genomics Institute, University of California; University of Miami; Dirección de Investigaciones, Fundación Huésped, Buenos Aires, Argentina.; Dirección de Investigaciones, Fundación Huésped, Buenos Aires, Argentina.; Instituto de Fisiología (IFIBYNE), Biología Molecular y Neurociencias (IFIBYNE), Buenos Aires, Argentina; Weill Cornell Medicine; University of Miami; CINIBA, Universidad Nacional de La Plata

**Keywords:** Kaposi’s Sarcoma, RNAseq, Transcriptome, Xena browser

## Abstract

Kaposi sarcoma (KS) is an AIDS-defining cancer and a significant global health challenge caused by KS-associated herpesvirus (KSHV). NGS-based approaches have profiled KS lesions in a minimal number of studies compared with other neoplastic diseases. Here we present a compiled and harmonized dataset of 131 KS and non-tumor cutaneous samples in the context of their predicted pathway activities, immune infiltrate, KSHV and HIV gene expression profiles, and their associated clinical data representing patient populations from Argentina, United States (USA), and Sub-Saharan Africa cohorts. RNA-seq data from 9 Argentinian KS lesions were generated and integrated with previously published datasets derived from the USA and sub-Saharan African cohorts from Tanzania, Zambia, and Uganda. An unsupervised analysis of 131 KS-related samples allowed us to identify four KS clusters based on their host and KSHV gene expression profiles, immune infiltrate, and the activity of specific signaling pathways. The compiled RNA-seq profile is shared with the research community through the UCSC Xena browser for further visualization, download, and analysis (https://kaposi.xenahubs.net/). These resources will allow biologists without bioinformatics knowledge to explore and correlate the host and viral transcriptome in a curated dataset of different KS RNA-seq-based cohorts, which can lead to novel biological insights and biomarker discovery.

## 1 INTRODUCTION

Kaposi sarcoma (KS) is a prominent AIDS-defining malignancy, and a critical global health challenge primarily linked to infection with KS-associated herpesvirus (KSHV) (Mesri et al., 2010; Dittmer and Damania 2016; Cesarman et al., 2019). KSHV contains a complex genome comprising over 80 genes, which are regulated to facilitate either latency with minimal viral expression or lytic replication, resulting in the production of new virions. KSHV can infect epithelial cells, endothelial cells, B cells, and, more recently, it has been found to infect neurons and mesenchymal stem cells (MSCs), further highlighting its diverse pathogenic potential (Mesri et al., 2010; Dittmer and Damania 2016; Cesarman et al., 2019; Naipauer and Mesri 2023; Lacunza et al., 2024).

Among the various forms of KS, the most aggressive variant is observed in individuals with HIV, known as acquired immunodeficiency syndrome-associated KS (AIDS-KS). This form is characterized by widespread dissemination, affecting the skin and visceral organs, including the gastrointestinal tract and lungs (Haverkos et al 1985). Moreover, in men who have sex with men (MSM), the HIV infection rate is 4.9–10.5%, and in transgender women (TGW), HIV reported prevalence is up to 58%. These two populations have disproportionately higher risks of developing KS caused by KSHV (Hutchison et al., 2018; AIDS-defining Cancer Project Working Group 2017).

NGS-based transcriptomic analyses of KS have revealed significant molecular insights into the pathogenesis of the disease. For example, Tso et al. (2018) reported alterations in glucose and lipid metabolism, with changes in genes associated with metabolic disorder pathways. A recent study further stratified HIV + KS lesions into two molecular subtypes—one characterized by endothelial and proliferative features and the other enriched in inflammatory transcripts (Moorad et al., 2023). Additionally, profiling KS lesions from different anatomical sites has revealed variations in immune composition, angiogenic factors, and KSHV gene expression, underscoring the role of the tumor microenvironment in disease progression (Ramaswami et al., 2023). Unsupervised clustering of KSHV gene expression has also identified distinct tumor groups with varying latent and lytic viral profiles (Tso et al., 2018; Rose et al., 2018; Lidenge et al., 2020; Lacunza et al., 2024). Furthermore, morphologically distinct KS tumors from the same individual exhibit similar KSHV gene expression patterns, suggesting that viral activation is influenced by the local microenvironment and host immune response (Rose et al., 2018). This molecular heterogeneity emphasizes the need for further studies to develop predictive biomarkers and optimize therapeutic strategies for KS.

Most of these RNAseq studies come from subjects in sub-Sahara Africa where KSHV seroprevalence is notably high, with over 50% of the population exhibiting detectable anti-KSHV antibodies (Uldrik et al., 2011; Damania and Dittmer 2023). This raises the need for a broader integrative analysis including diverse cohorts to capture the global spectrum of KS pathology.

This study aimed to integrate RNAseq-based gene expression profiles from four distinct cohorts of KS covering North America, South American, and sub-Saharan Africa to enhance our understanding of the disease. Additionally, we intend to share these valuable datasets with the research community through the UCSC Xena resource, promoting further exploration and discovery in the field of KS research.

## 2 MATERIALS AND METHODS

### 2.1 Sample Collection and RNA Sequencing

Nine KS skin lesions were collected within an Argentinian study that included men at birth with or without HIV, recruited at Fundación Huésped and Hospitals network as part of our collaborative U54 consortium (Miami CFAR SCCC - Argentina Consortium for Research and Training in virally induced AIDS-Malignancies). Participants were > 18 years old (median: 42 years; range: 34–62 years). Approval of the protocol and study-related documents was obtained from the Ethics Committee. All participants included in this study signed informed consent before undergoing any study procedures.

Total RNA was isolated from KS samples preserved in RNAlater using the miRNeasy Tissue/Cells Advanced Kits (Qiagen) following standard manufacturer’s protocol. RNA concentration and integrity were measured on an Agilent 2100 Bioanalyzer (Agilent Technologies). RNA samples with RNA integrity number (RIN) over 5 were considered for RNA sequencing. The RNA samples were processed for directional RNA-seq library construction using the Illumina Total RNA Prep with Ribo-Zero Plus library preparation kit according to the manufacturer’s protocol. We performed 101 nt paired-end sequencing using an Illumina Novaseq 6000 platform and obtained ~ 60 million clusters per sample with 92% >Q30. The RNAseq raw data has been submitted to NCBI GEO database with accession number GSE271303.

### 2.2 RNAseq data pre-processing and integration of Kaposi’s Sarcoma cohorts

RNAseq raw data were retrieved from GEO/SRA using the SRA Toolkit (https://github.com/ncbi/sratools) from GSE147704 (Tanzania & Zambia cohort: KS = 24, Control = 24, and Normal = 3), GSE241095 (USA cohort: KS = 10 and Control = 10) and SRP486827 (Uganda cohort: KS = 51). The raw short-read sequences generated from the Argentinian KS samples and retrieved from GEO/SRA were quality-checked and trimmed to remove adapters and low-quality bases using the Rfastp R/Bioconductor package. The preprocessed reads were then aligned and mapped to the human genome reference GRCh38 using the Subread aligner algorithm provided by the Rsubread R/Bioconductor package. The remaining non-human reads were aligned to the KSHV reference sequence NC_009333. The KSHV reference genome used in our study for read sequence alignment and mapping belongs to the Human herpesvirus 8 strain GK18. It provides a valuable complete genome for global transcriptome analysis, but it does not capture the full extent of KSHV’s genetic diversity, including the highly diverse K1 and K15 subtypes present in sub-Saharan populations. The aligned reads (BAM files) from each sample were used to calculate gene expression abundance at the whole-genome level using the featureCounts function provided by the Rsubread package.

The raw read counts for each cohort were integrated, and batch effects were diagnosed using the BatchQC R/Bioconductor package (Manimaran et al., 2016), followed by batch effects adjustment with the ComBat-seq algorithm from the “sva” R/Bioconductor package (Zhang et al., 2020). Briefly, ComBat-seq algorithm implements a negative binomial regression model to estimate batch effects based on the count matrix obtained for each RNA-seq study allowing us to include the condition variable (control or KS) as covariates in the regression model behind this method to account for the impact of batch and retaining biological signal.

### 2.3 KS and KSHV transcriptome analysis of the compiled dataset

The transcriptome profile of the compiled batch-effect adjusted KS dataset consists of 131 KS and non-tumor cutaneous samples and their associated metadata. To identify differentially expressed genes between KS and controls or between KS groups (e.g.: from HIV + vs HIV− or with or without ART, etc.), we computed fold changes and adjusted p-values using the edgeR R/Bioconductor package based on the normalized log2-based count per million values. Genes showing a log-fold change greater than 1 and an adjusted p-value below 0.05 were considered significantly differentially expressed. Functional enrichment analysis and Gene Set Variation Analysis (GSVA) of differentially expressed genes were performed with the clusterProfiler and GSVA R packages. Tumor immune cell infiltration scores were estimated with the ABIS algorithm and the MCP-counter tool from the immunedeconv (https://github.com/omnideconv/immunedeconv) and MCPcounter (https://github.com/ebecht/MCPcounter) R packages, respectively, on normalized count matrices. The scores, based on transcriptomic markers that are strongly, specifically, and stably expressed in a unique cell population, are proportional to the abundance of each population in the tumor, enabling intersample comparison and large cohort analyses (Becht et al., 2016). Unsupervised hierarchical clustering analysis and heatmaps representations were performed with the MultiExperimentViewer (MeV 4.9.0) software. To determine the optimal number of clusters, we applied Principal Component Analysis (PCA) alongside the NbClust package, which evaluates the number of clusters using 30 different indices(https://github.com/cran/NbClust/blob/master/R/NbClust.R).

The relationship between a categorical and a quantitative variable was assessed using the Wilcoxon rank-sum test or the Kruskal–Wallis test. Pearson correlation was used for two quantitative variables. P values were corrected for multiple testing using the Bonferroni or Benjamini–Hochberg methods.

### 2.4 Data sharing and visualization through a UCSC Xena hub

UCSC Xena is a high-performance visualization and analysis tool for both large public repositories and private datasets (Goldman et al., 2020). UCSC Xena has two components: the front-end Xena Browser and the back-end Xena Hubs. We have deployed a public Xena Hub (called KS-omics) to host and share the functional genomics data of the compiled KS dataset with the research community (https://kaposi.xenahubs.net/). Briefly, Xena Browser allows biologists without bioinformatics knowledge to explore data with a variety of visualizations and analytic tools.

## 3 RESULTS AND DISCUSSION

### 3.1 Integrative Analysis of Host and Viral Gene Expression Profiles in KS Using Multi-Cohort RNA Sequencing

**Figure 1:**
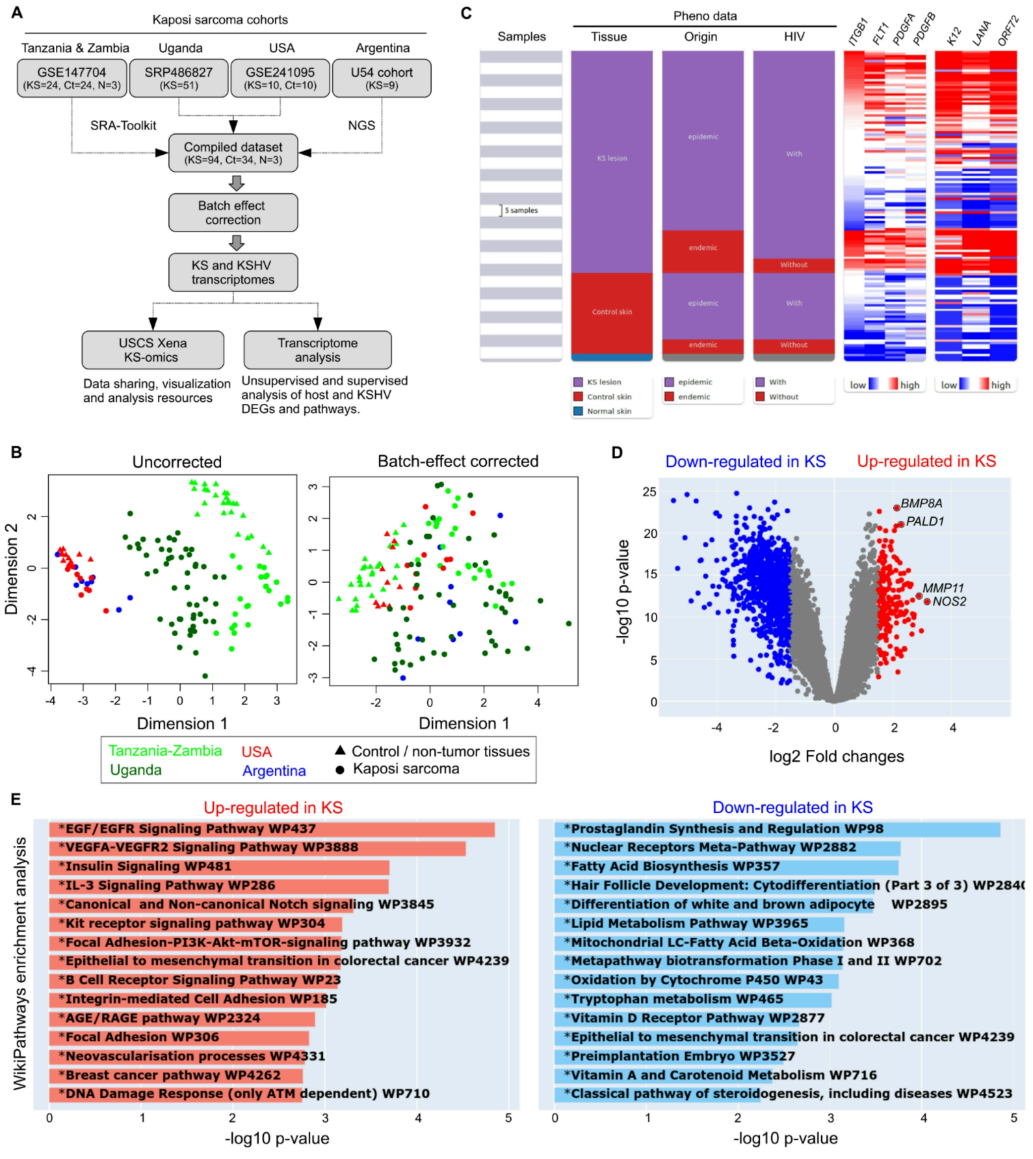
Compiled KS dataset of 131 KS and matched non-tumor tissues. **A.** Diagram of the Strategy followed to obtain the compiled KS dataset. **B.** Multidimensional scaling plot of the non-adjusted (left) and batch-effect adjusted (right) gene expression data of KS (circle) and matched non-tumor tissues (triangle) among cohorts. **C.** An example Xena Browser Visual Spreadsheet examining the gene expression profiles of selected humans (*ITGB1, FLT1, PDGFA* and *PDGFB*) and KSHV genes (*K12, LANA, ORF72*) among the compiled KS dataset in the context of their phenotypic data (https://kaposi.xenahubs.net/). Gene expression data is colored red to blue for high to low expression respectively. UCSC Xena browser provides analytic tools to correlate the human and KSHV gene expression levels as well as to identify differentially expressed genes-based groups defined by the user (*e.g*. KS lesions vs. control skin). **D.** Representative volcano plot of differentially expressed genes detected between KS and control samples using UCSC Xena browser. **E.** Functional enrichment analysis of differentially expressed genes with Xena.

To perform an integrative analysis of host and viral gene expression profiles in KS, we combined 94 KS with 37 non-tumor cutaneous cases obtained from four cohorts, one of them generated by us and three available in a public database (Figure 1A). Clinical variables considered for the study included age, ART treatment, sex at birth, HIV status, origin (endemic/epidemic), and KS morphotype. To generate a homogeneous, uniformly curated, and preprocessed dataset, the RNAseq raw data was directly retrieved from GEO/SRA using the SRA Toolkit and equally preprocessed by our bioinformatics pipeline. Additionally, raw read count matrices from each cohort were integrated. Variation analysis (Figure **S1**) showed that batch effects explained a larger proportion of variation than the condition (control or KS), highlighting the need for batch correction. Batch effects were then adjusted using the ComBat-seq algorithm (Figure **S1**). Importantly, the transcriptomic profile of the four independent KS cohorts were generated using Illumina sequencing platforms (Table 1).

Figure 1B shows the scatter plot of cases projected on the first two dimensions in unadjusted data, and in data adjusted by ComBat-seq. We observed a strong batch effect in the unadjusted data, which was well addressed by ComBat-seq. A favorable adjustment would pool control/non-tumor cutaneous samples from the cohorts (batches), while keeping all KS lesions separated from the controls and from each other. In the multidimensional plot of ComBat-seq adjusted data, we observed the expected pattern of data if there were no batch effects, in which the control/normal cases are clustered together, while the KS cases from four cohorts are scattered at different locations. These results suggest a successful adjustment of batch effect from ComBat-seq.

UCSC Xena is a high-performance visualization and analysis tool for both large public repositories and private datasets (Goldman et al., 2020). In this sense, we have implemented a public cloud-based backend Xena Hubs hosting the human and KSHV batch effect adjusted gene expression, immune and pathway activity profiles as well as their associated phenotypic data obtained from the four KS cohorts (https://kaposi.xenahubs.net/). The frontend Xena Browser provides a wide variety of visualizations and analyses including scatter plots, bar graphs, statistical tests, genomic signatures, as well as a unique Visual Spreadsheet view (Figure 1C). The Xena Visual Spreadsheet was designed to enable and enhance integration across diverse data modalities, providing researchers with a more biologically complete understanding of genomic events and tumor biology. The UCSC Xena browser enables users to explore functional genomic datasets for correlations between genomic and phenotypic variables. It also allows for differential expression analysis between user-defined sample groups (Figure 1D) and functional enrichment analysis of differentially expressed genes (Figure 1E).

### 3.2 Unsupervised clustering reveals distinct transcriptomic and immune profiles in KS lesions.

To characterize the transcriptomic, immune, and functional profiles of the samples, we employed an unsupervised clustering approach. We defined clusters based on the entire transcriptome (host and KSHV). This approach allows us to dissect the contributions of both host and viral factors to the overall expression landscape, providing a comprehensive understanding of the interplay between the host’s immune response and viral activity in KS.

**Figure 2:**
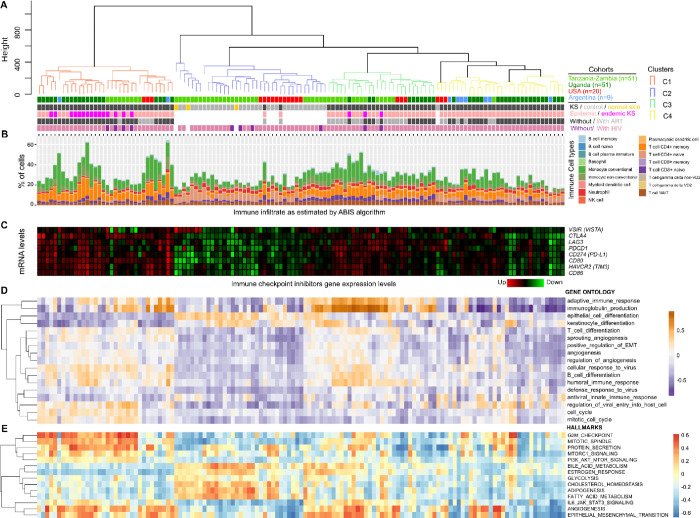
Transcriptomic, Immune and Functional Profiling of Kaposi Sarcoma Lesions. **A.** Clustering results reveal distinct groups of KS lesions, with endemic lesions primarily in Cluster C1, while epidemic lesions are distributed across the three clusters. **B.** Immune profiling using the ABIS algorithm demonstrates a higher immune cell infiltrate in KS lesions compared to controls. **C.** Transcriptomic expression levels of immune checkpoint inhibitors (ICIs) indicate that Clusters C1 and C3 exhibit the highest expression levels. **D.** Pathway activity analysis using Gene Ontology reveals reduced epithelial differentiation and increased immune activity in KS lesions, particularly in Clusters C1 and C3. Cluster C4 shows lower immune activity but a strong innate antiviral response. **E.** Pathway activity analysis using Hallmarks reveals reduced metabolic processes in KS lesions compared to controls, along with increased proliferative activity, angiogenesis, and PI3K/Akt/mTOR signaling, particularly in Clusters C1 and C3.

The unsupervised analysis revealed a clear distinction between KS lesions and non-tumor controls (Figure 2A), underscoring the impact of KSHV on the host transcriptome (Tso et al., 2018). To further validate the optimal number of clusters, we applied the NbClust package. The majority rule suggested either 2 or 4 clusters as the most likely options. Considering this, along with the structure observed in the dendrogram and PCA analysis, we determined that 4 clusters were the most appropriate choice for our data (Figure **S2**) We identified at least three distinct groups of lesions, designated as clusters 1 (C1), 3 (C3), and 4 (C4). Uninvolved skin from KS patients and skin from non-KS subjects were classified within the control cluster (C2), which comprised two subclusters and included two KS cases (Figure 2A). Cluster C1 included most endemic tumors, along with some epidemic cases, while clusters C3 and C4 were primarily composed of epidemic lesions (p<0.001; Data **S1**). Importantly, no significant associations were observed between lesion clusters and HIV status or ART treatment (Data **S1**). However, a notable association with KS morphotype was found, with cluster C4 exhibiting a higher percentage of macular lesions (p<0.01; Data **S1**).

We defined the immune fraction profile using the ABIS algorithm, which demonstrated a significant immune infiltrate enrichment in the lesions compared to the controls (Figure 2B). Clusters C1 and C3 showed the highest percentage of immune fractions, indicating a greater infiltrate compared to clusters C2 and C4. Cluster C1 was characterized by a significant enrichment of memory CD4 T lymphocytes, whereas naive CD4 T cells were predominant in the control group (C2) (Figure **S3**). In contrast, cluster C3 exhibited a notable enrichment of plasmablasts and memory B cells (Figure **S3**). Additionally, all three lesion clusters showed enrichment in CD8 memory T cells compared to the control group (Figure **S3**).

To strengthen the analysis, we examined the composition of the sample microenvironment using the microenvironment cell populations (MCP)-counter method (Becht et al., 2016). The cell composition varied significantly between clusters (Figure **S3**). Cluster C1 displayed elevated expression of genes specific to immune populations, including T cells, CD8+ T cells, natural killer (NK) cells, and cytotoxic lymphocytes. However, it was primarily characterized by a significant upregulation of endothelial cell-related genes (Figure **S3**). Cluster C3 showed elevated expression of most immune cell populations, with B lineage signatures and T cells as key determinants. Cluster C4 displayed a generally low immune profile, similar to the control cluster (C2), but differed by a higher expression of endothelial cell markers (Figure **S3**).

These results complemented the findings by Tso et al., who reported a significant infiltrate of immune cells in the lesions, primarily of B cells, although T cells were not significantly present. However, they predicted the activation of numerous chemokines that play an important role in the recruitment of T cells (Tso et al., 2018). Additionally, Lidenge et al. have shown that, despite the upregulation of chemokines like CxCL-9, immune cells, including CD4+ T cells, CD8+ T cells, and NK cells, are not co-localized with KSHV-infected regions (Lidenge et al., 2020). This discrepancy highlights the differences between transcriptomic-based immune profiling and protein-level detection, suggesting that immune cells may be recruited to the tumor microenvironment but may not be localized to KSHV-infected areas. In addition, the marginal tissues captured in biopsies likely contain immune populations that are present but infiltrate the tumor at low frequencies.

Given the increasing relevance of immune checkpoint inhibitors (ICIs) in the treatment of KS, we evaluated the transcriptomic expression levels of a set of ICIs. A distinct pattern of expression levels was observed, correlating with the infiltrate (Figure 2C). In this context, clusters C1 and C3 were the most enriched in these genes. These results, while needing further validation, along with the immune profile of the clusters, are relevant in the context of the KS microenvironment and immune checkpoint ICIs immunotherapies. They are also consistent with previous studies. For instance, Petitprez et al. (2018) established an immune-based classification of sarcoma immune classes (SICs) and demonstrated that the immune-high group, characterized by the presence of B cell-rich lineage, exhibited a strong response to PD1 immunotherapy. The tumor microenvironment in KS is known to be composed of activated B cells and tumor-associated macrophages (Joest et al., 2020). More recently, it has been shown that KS, along with other sarcoma subtypes, displayed the highest response rates to ICIs and longest survival, with PD-L1 expression of ≥1% being associated with increased response to ICIs (Lee et al., 2024).

### 3.3 Pathways activity analysis.

The pathway activity analysis based on Gene Ontology (GO) and Hallmarks revealed a reduction in epithelial differentiation, including keratinocyte differentiation and epithelial-mesenchymal transition (EMT), in lesions compared to controls (Figure 2D). Additionally, decreases in metabolic processes such as lipid metabolism, cholesterol homeostasis, and response to estrogen were observed (Figure 2E). This finding aligns with previous studies demonstrating that KSHV plays a significant role in cellular transformation and metabolic reprogramming, both of which are key factors in the pathogenesis of KS (Zhu et al., 2014; Tso et al., 2018). Moreover, the impaired EMT is particularly important in KS, as it promotes the formation of new blood vessels and supports tumor growth, emphasizing the complex relationship between these processes in the disease (Naipauer and Mesri 2023; Lacunza et al., 2024).

Furthermore, the analysis revealed higher levels of immune pathways—such as adaptive immune responses, T cell differentiation, and B cell differentiation—in the lesions of clusters C1 and C3 compared to cluster C4. KS lesions in cluster C3 exhibited more pronounced humoral immune related pathways than those in cluster C1, which corresponds with its higher proportion of B cells (Figure 2D, Figure **S3**). Since KSHV has a known tropism for B cells and can infect them, leading to lymphoproliferative lesions, cluster C3, enriched in B cell lineages, may reflect this interaction (Rappocciolo et al., 2008; Ballon et al., 2011; Nicol et al., 2016). However, the transcriptomic data does not clarify whether these B cells are directly infected by KSHV or are merely part of the recruited immune response.

Lesions in cluster C4 displayed a pattern more similar to non-tumor controls, showing lower enrichment levels across most pathways compared to clusters C1 and C3 (Figure 2D). However, these lesions exhibited enrichment in pathways related to innate antiviral responses, whereas tumors in clusters C1 and C3 were associated with broader and more robust immune responses (Figure 2D).

Cluster C1 exhibited lesions with a molecular pattern of pathway activation characteristic of KS, marked by increased proliferative activity (e.g., cell cycle, mitotic spindle) and activation of the PI3K/Akt/mTOR and IL6/JAK/STAT3 pathways (Figure 2D, E). Both clusters C1 and C3 also displayed angiogenic activity (Figure 2D, E). These differences in immune and pathways profiling may have important implications for disease progression and patient response.

### 3.4 Gene expression profiling analysis.

To identify the representative genes or signatures associated with the functional processes in each lesion cluster, we conducted differential expression analysis, comparing controls (C2) with lesions (C1, C3, and C4), as well as among the lesion groups themselves. Cluster C1 exhibited the highest number of dysregulated genes compared to non-tumor samples, with the majority of KSHV genes expressed (Figure **S4**, Table **S1**). KSHV genes *K15, K13, ORF72, ORF75, vIRF-2,* and *vIRF-4* were among the top upregulated genes. Among the numerous affected host genes, those linked to extracellular matrix remodeling stood out, including several metalloproteinases and collagens (e.g *MMP9–17, ADAMTS54-S7, COL4A1*), along with significant genes such as *ITGB1, FLT1, FLT2, PDGFA, PDGFB, AKT3, ANGPT2, CXCL10, ROCK2, IFNG,* and *CXCR4*, all of which are associated with processes relevant to KS, such as endothelial differentiation, angiogenesis, the PI3K/Akt pathway, and cytokine/chemokine cascades (Cesarman et al., 2019). Additionally, several genes related to cell proliferation (e.g. *PLK1, BUB1B, TTK*) and T cell lymphocytes (e.g. CD86, CD274, CCR7) were also upregulated. There was also a marked decrease in the expression of several genes linked to protein biosynthesis (ribosomal proteins), cellular respiration (e.g. *NDUFA1, NDUFA3, CYC1*), and lipid metabolism (*ADIPOQ, ACOX1, ACOX2, CYP4B1*) This underscores the substantial impact of KSHV on host cell metabolism, particularly under hypoxic conditions (Delgado et al., 2010; Singh et al., 2018; Méndez-Solís et al., 2021).

When comparing cluster C3 with controls (C2), a similar trend emerged, with a high expression of KSHV genes and a notable impact on host genes, though to a lesser extent than in cluster C1 (Figure **S4**, Table **S1**). In cluster C3, there was a prominent enrichment of genes associated with lymphocyte activation and proliferation (e.g., *CD80, ITK, CD84, CD7, CD28, CD247, TLR9, LYN, CD22*). Cluster C4 also exhibited expression of most KSHV genes when compared to non-tumor controls; however, the host gene enrichment was primarily linked to the viral infection response (e.g., *IFIT1, IFIT2, IFIT3, IRF1, IRF7, IF16*) (Table **S1**).

The comparison between lesion clusters revealed distinct differences. In the cluster C1 vs. cluster C3 analysis, cluster C1 exhibits gene expression profiles more like KS tumors, specifically angiogenic genes (e.g. *TX1, ANGPTL3, PROX1, SCUBE1, KDR, ROBO4, PDGFB, SERPINA5*), mitotic genes (e.g. *CENPE, CDK1, KIF3C, BRCA2, TOP2A*, etc.), and remodeling matrix components including metalloproteinases (Lacunza et al., 2024). Conversely, cluster C3 was distinguished by greater differentiation (higher keratin expression) and increased metabolic activity, evidenced by a higher expression of lipid metabolism-related genes and xenobiotic metabolism genes with activation of numerous cytochrome P450 genes (Table **S1**).

The comparison between clusters C1 and C4 reveals distinct metabolic and immune response profiles in the lesions. Cluster C4 shows elevated expression of oxidative and xenobiotic metabolism genes, along with ERBB signaling genes (e.g., *ERBB2, ERBB3, ERBB4, EGFR, EREG*), suggesting an adaptive response to environmental stressors and a focus on cellular survival and proliferation (Table **S1**). This points to potential metabolic reprogramming that could support tumorigenesis. The ERBB family of kinases has recently been implicated in regulating the KSHV latent-to-lytic replication switch, emphasizing their role in KSHV-related pathogenesis (Olson et al., 2023). In contrast, cluster C1 was marked by a strong activation of immune-related genes, particularly those associated with lymphocyte activation and inflammatory responses (e.g., *CXCL8, CXCL13, CXCL2, IL6, IFNG, IL10, CCR5, CXCR4*), indicating a robust immune environment potentially engaged in combating the tumor (Table **S1**).

Additionally, the comparison of clusters C3 and C4 revealed a lower number of differentially expressed genes (Figure **S2**). C3 exhibited higher expression of genes linked to T and B cell activation (e.g., *CD84, CD86, CD70, CD79A, CD79B, IL10*), while C4 showed increased expression of genes involved in antiviral responses (e.g., *IFI44, IFIH1, IFI27, RSAD2, XAF1, OAS1, OAS2, OAS3*) (Table **S1**).

In summary, the transcriptomic and functional analysis identifies three distinct groups of KS. Cluster C1, primarily composed of endemic cases with some epidemic ones, represents tumors characterized by poorly differentiated, endothelial, and proliferative phenotypes. This cluster also showed enrichment of the PI3K/mTOR pathway and a strong presence of memory CD4 T lymphocytes in the immune fraction. Cluster C3, composed of predominantly epidemic cases, showed greater metabolic activity and was enriched in B cells. In contrast, cluster C4 had the least immune infiltration and highest differentiation, with strong activation of antiviral defense genes and the macular morphotype, typical of early-stage KS (Cesarman et al., 2019).

Overall, consistent with the findings of Lidenge et al. (2020), which showed that endemic and epidemic KS transcriptomes are highly similar, with enhanced dysregulation of tumorigenesis and immune functions in endemic KS, we observed a segregation between endemic (C1) and epidemic (C3 and C4) cases. This distinction was driven by the magnitude and the specific subsets of expression changes. C1 (primarily endemic) displayed stronger activation of angiogenesis, endothelial differentiation, immune-related genes, cell proliferation, and tumorigenic pathways such as PI3K/Akt/mTOR. In contrast, C3 (epidemic) exhibited higher metabolic activity, with increased expression of genes related to lipid and xenobiotic metabolism, while C4 showed features typical of an early-stage viral response, including activation of antiviral defense pathways. These differences highlight functional divergence between C1, C3, and C4, supporting the clustering of endemic and epidemic KS lesions based on distinct immune and metabolic signatures.

### 3.5 KSHV transcriptome analysis.

**Figure 3:**
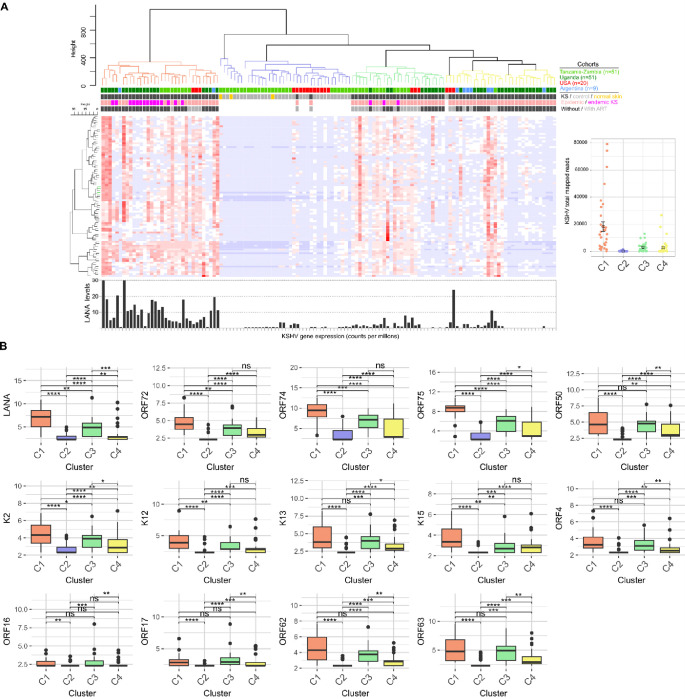
KSHV Transcriptomic Analysis Across Kaposi Sarcoma Lesion Clusters **A.** Heatmap visualization of the expression levels of 85 KSHV genes across control and lesion clusters. Gene expression data is colored red to blue for high to low expression respectively. The bar plot at the bottom displays LANA levels based on mapped reads. The error bar plot on the right illustrates the total KSHV mapped reads in each cluster, highlighting significant differences in cluster C1 compared to clusters C3 and C4 (p<0.01). **B.** Bar plots displaying the expression of key KSHV genes that are differentially expressed between clusters. * p <0.05; ** p<0.01; *** p<0.001; **** p<0.0001.

To evaluate the relationship between the clusters identified from the whole transcriptome and KSHV expression levels while minimizing variability from host gene expression, we chose to analyze the KSHV transcriptome alone between clusters. This approach clarified the viral contributions to the disease and helped to determine if different viral expressions correlate with the observed clusters. First, it is important to note that there were no significant differences in overall KSHV expression levels (measured by total mapped KSHV reads, TMKR) across the different cohort populations (Argentina, Tanzania/Zambia, and Uganda; p>0.05; Figure **S5**). However, when comparing the clusters, cluster C1 contained tumors with the highest levels of KSHV expression, as indicated by the TMKR, compared to clusters C3 and C4 (p<0.01; Figure 3A). This difference was further supported by the elevated levels of LANA in cluster C1 (Figure 3A). The analysis of differentially expressed genes among the lesion clusters confirmed that cluster C1 exhibited the highest expression levels of genes such as *ORF72, ORF73* (*LANA*), *ORF74, ORF75*, as well as *K12, K13,* and *K15*, which were among the most highly expressed transcripts across all clusters. These genes have been implicated in various functions, including immune evasion, angiogenesis, cell proliferation, and apoptosis regulation. LANA is known to stabilize the viral genome within host cells, facilitating persistence and contributing to tumorigenesis (Uppal et al., 2014; Nakajima et al; 2024). *ORF74* is a viral GPCR that binds various human chemokines (*CXCL1, CXCL8*) contributing to immune evasion and consequently viral dissemination (de Munnik et al., 2015; Medina et al., 2020). Similarly, *K12* and *K13* play roles in modulating host immune responses (Liang et al., 2008), suggesting that the elevated expression of these genes in cluster C1 may correlate with more aggressive tumor behavior or enhanced viral persistence. Although no significant differences were observed in the total mapped reads between clusters C3 and C4 (Figure 3A), these clusters were differentiated by the expression of specific genes, including LANA and RTA (*ORF50*). Additionally, lytic genes such as the viral Bcl-2 (*ORF16*), which inhibits apoptosis, the protease (*ORF17*) associated with capsid maturation, and the gene expression modulator (*ORF18*) were all more abundantly expressed in C3 compared to C4 (Figure 3B; Table **S2**).This suggests that KSHV is contributing to the intrinsic transcriptomic differences among these clusters of epidemic tumors, potentially influencing their pathogenic behavior and responses to treatment. The distinct expression profiles of these genes highlight the complex interplay between viral replication and host cell dynamics in KS.

We conducted further correlation analyses to examine the relationship between viral tissue load (quantified via TMKR) and KS morphotypes. The macular group exhibited a significantly lower viral load compared to the plaque and nodular groups (Figure **S5**). Although no significant differences were found between the plaque, nodular, and fungating groups, an increasing trend in mean TMKR values was observed from macular to fungating lesions (Figure **S5**). These findings correlate with the observation that cluster C4, primarily composed of macular lesions, exhibited lower viral load, consistent with the early stage of these lesions. This suggests an association between viral load and lesion stage in KSHV-driven tumorigenesis. We also explored the relationship between immune infiltration patterns and viral loads across clusters (Figure **S5**). Linear regression analysis revealed that higher KSHV loads were associated with a decrease in naïve CD4+ T cells and an increase in memory CD4+ T cells, particularly in Cluster C1. A similar trend, though weaker, was observed for CD8+ T cells, with memory CD8+ T cells showing a positive association with KSHV load. No significant associations were found between KSHV load and B cell subsets, suggesting that B cell enrichment in cluster C3 may be an intrinsic feature of these lesions, independent of KSHV load.

While our study provides valuable insights into the molecular diversity of KS, several limitations should be acknowledged. First, RNA-seq data, while powerful for transcriptomic profiling, may not fully capture the presence and spatial distribution of immune cells within the tumor microenvironment. Immune profile algorithms infer immune cell composition based on gene expression rather than direct cellular localization, which may not reflect the actual immune cell presence in KS lesions. Additionally, although our findings align with and expand upon previous transcriptomic studies that identified varying KS subtypes based on proliferative, angiogenic, or metabolic pathways, the classification of KS lesions remains complex. The number of subtypes identified across studies has ranged from two to five, highlighting the heterogeneity of KS tumors (Tso et al., 2018; Rose et al., 2018; Lidenge et al., 2020; Ramaswami et al., 2023; Moorad et al., 2023). Despite these limitations, our study’s strength lies in integrating diverse cohorts, providing a more comprehensive view of KS pathology across different populations. Further validation, including spatial transcriptomics, will be necessary to refine our understanding of KS molecular landscapes and their therapeutic implications.

## 4 CONCLUSIONS

In summary, this is the first study to integrate transcriptomic profiles of various cohorts of KS. The RNA-seq profile generated from the Argentinian KS samples compiled with previously published datasets as well as their associated metadata and predicted pathway activities, immune infiltrate, host and viral gene expression profiles are shared with the research community through the development of a KS-omics resource leveraging UCSC Xena browser. This resource will allow biologists without bioinformatics knowledge to explore and correlate the host and viral transcriptomic profiles in a compiled and harmonized dataset to develop novel biological insights and discover biomarkers.

Our comprehensive analysis enabled us to define and characterize three distinct lesion groups that reflect both the biology of the virus and the intrinsic molecular characteristics of the lesions. Notably, while endemic lesions were primarily concentrated in cluster C1, epidemic lesions were distributed across all three clusters.

Epidemic lesions in clusters C3 and C4 did not show significant differences in overall KSHV expression but differed in their intrinsic functional molecular characteristics. Cluster C3 exhibited a high level of B cell infiltration and ICIs expression, while cluster C4 was characterized by an enrichment of genes involved in early response against viral infection. Whether these different clusters imply differences in treatment approaches or response, it still needs to be explored.

Thus, we defined three groups of KS: endothelial, proliferative and well-defined KS tumors (C1); B-cell-enriched and metabolic tumors (C3); and less immunogenic, early-stage KS tumors (C4). Overall, our findings provide valuable insights into the molecular diversity of KS and underscore the potential for tailored therapeutic strategies based on lesion characteristics.

## Figures and Tables

**Table 1.** Characteristics of the study subjects included in each KS cohort.

Characteristics	Tanzania / Zambia	Uganda	USA	Argentina
	GSE147704	SRP486827	GSE241095	GSE271303

# of Cases	24 KS	51 KS	10 KS	9 KS
	24 Controls		10 Controls	
	3 Normal skin			

Age (median, IQR)	33 (29, 42)	35 (26, 46)	43 (34, 49)	42 (34, 50)

Sex at birth				
Male	16	44	10	9
Female	8	5	0	0
n/a	-	2	-	-

Race				
White	0	0	2 (20%)	0
Black	24 (100%)	51 (100%)	4 (40%)	0
Hispanic	0	0	4 (40%)	9 (100)

HIV characteristics				
HIV co-infection	18 (75%)	51 (100%)	9 (90%)	9 (90%)
CD4 T-cell count (cells/ul)	na	na	39	144
HIV Viral load (cps/mL)	12000	na	74000	125000
On ART at biopsy	18 (75%)	0 (0%)	10 (100%)	3 (33%)

## Data Availability

The raw data have been submitted to NCBI GEO database with accession number GSE271303. The rest of the data are available from the corresponding author upon reasonable request.
